# Regulation of TSC2 lysosome translocation and mitochondrial turnover by TSC2 acetylation status

**DOI:** 10.1038/s41598-024-63525-7

**Published:** 2024-05-31

**Authors:** Patricia Marqués, Jesús Burillo, Carlos González-Blanco, Beatriz Jiménez, Gema García, Ana García-Aguilar, Sarai Iglesias-Fortes, Ángela Lockwood, Carlos Guillén

**Affiliations:** 1 Department of Biochemistry and Molecular Biology, Faculty of Pharmacy, Complutense University of Madrid, Madrid, Spain; 2 CIBER of Diabetes and Associated Metabolic Disorders, Instituto de Salud Carlos III, Madrid, Spain; 3 Department of Biochemistry and Molecular Biology, Faculty of Pharmacy, Complutense University of Madrid, IdISSC, Madrid, Spain; 4 Department of Pharmacology, Pharmacognosy and Botany, Faculty of Pharmacy, Complutense University of Madrid, Madrid, Spain; 5 P2022/BMD-7227, MOIR-ACTOME-CM, Dirección General de Investigación e Innovación Tecnológica (DGIIT), Consejería de Educación y Universidades, Comunidad de Madrid, Madrid, Spain

**Keywords:** TSC2, Acetylation, Lysosome, mTORC1, Mitophagy, Pancreatic β cells, Biochemistry, Endocrine system and metabolic diseases

## Abstract

Sirtuin1 (SIRT1) activity decreases the tuberous sclerosis complex 2 (TSC2) lysine acetylation status, inhibiting the mechanistic target of rapamycin complex 1 (mTORC1) signalling and concomitantly, activating autophagy. This study analyzes the role of TSC2 acetylation levels in its translocation to the lysosome and the mitochondrial turnover in both mouse embryonic fibroblast (MEF) and in mouse insulinoma cells (MIN6) as a model of pancreatic β cells. Resveratrol (RESV), an activator of SIRT1 activity, promotes TSC2 deacetylation and its translocation to the lysosome, inhibiting mTORC1 activity. An improvement in mitochondrial turnover was also observed in cells treated with RESV, associated with an increase in the fissioned mitochondria, positive autophagic and mitophagic fluxes and an enhancement of mitochondrial biogenesis. This study proves that TSC2 in its deacetylated form is essential for regulating mTORC1 signalling and the maintenance of the mitochondrial quality control, which is involved in the homeostasis of pancreatic beta cells and prevents from several metabolic disorders such as Type 2 Diabetes Mellitus.

## Introduction

Eukaryotic cells need to regulate cell growth by the presence of growth factors and a correct sensing in nutrients such as glucose and aminoacids^1,2^. A modification in any of these two factors, generates a dysfunction in cell maintenance and is associated with different diseases such as cancer, type 2 diabetes mellitus (T2DM) and aging^3^. One of the main controllers of cell size and cell proliferation is the mechanistic target of rapamycin complex 1 (mTORC1). In fact, the hyperactivation of mTORC1 associated with a concomitant increase in glucose levels is observed in different pathologies such as T2DM^4^. In addition, in pancreatic β cells, the induction of mTORC1 in the absence of nutrients, such as glucose, triggers the apoptotic pathways^5^. Paradoxically, a chronic induction of AMPK signalling pathway occurs in these cells in vitro*,* which also stimulates apoptosis, being the autophagy machinery a protective mechanism to bypass the energetic stress induced^6^. Then, in all the cells and, especially in pancreatic β cells, which are responsible for the metabolic control of the organism and the energy homeostasis, it is necessary to maintain a correct balance between anabolic and catabolic pathways.

mTORC1 is essential in the coordination of growth factors with the energy status of the cell. The two major regulators of mTORC1 activation are two groups of GTPases belonging to the Ras superfamily, including Rheb and the Rag GTPases^7^. Rag GTPases are activated in response to aminoacids^8^. This group of proteins acts in dimers and Rag A or B associates with Rag C or D. When aminoacids are present, the ratio between RagA/B-GTP versus RagA/B-GDP is altered through the guanine exchange factor (GEF) activity of another complex called Ragulator^9^. Rheb protein acts as a monomer and is the other protein involved in the activation of mTORC1 signalling pathway. This protein switches between a GTP-bound form and a GDP-bound form, depending on the GTPase activity (GAP activity) of another protein called tuberous sclerosis complex 2 (TSC2)^10^. TSC2 forms a complex with two other components, TSC1 and TBC1D7^11–13^, acting as the main regulators of mTORC1 activation. TSC2 can be regulated by different post-translational modifications such as phosphorylation, which activates or inhibits its endogenous GAP activity towards Rheb. TSC2 phosphorylation is controlled by diverse kinases such as AKT, MEK/ERK and many others, stimulated mainly by growth factors such as insulin^14,15^. In addition, TSC2 is regulated by other groups of protein kinases that are activated under stress conditions including hypoxia and glucose deprivation, through REDD1 and AMPK activation respectively^16,17^. Several years ago, our group described that TSC2 lysine acetylation induces mTORC1 activation as well. Specifically, acetyl-TSC2 facilitates its association with ubiquitin and its degradation, facilitating mTORC1 activation^18^.

It is well known that mTOR can shuttle from a cytosolic location inside the cell, under basal conditions, to the lysosomal membrane, where it is activated after several stimuli^19^. Furthermore, TSC2 can also be recruited to the lysosome under different stress situations. For instance, after insulin stimulation, Akt phosphorylates TSC2 and dissociates it from the lysosome, favoring mTORC1 activation, by prevention of Rheb inactivation^20^. The regulation of TSC2 translocation to the lysosomal membrane has been observed by either aminoacid deprivation^21^ or glucose deprivation^6^ in different cell types, pointing to an opposite regulation between mTOR and TSC2 recruitment to the lysosome.

Autophagy is a catabolic process which is regulated by multiple stimuli. It is essential in the maintenance of cell homeostasis in organisms from yeast to humans^22^. This mechanism involves the generation of a double-membrane structure, which is known as autophagosome, that will engulf all the components that are going to be degraded. Then, the autophagosome fuses with the lysosome, and the content will be digested. Very interestingly, all the organelles inside the cells are controlled by a specific autophagic mechanism, including ER-phagy, nucleophagy, ribophagy and mitophagy among others^23^. Due to the essential role of mitochondria in pancreatic β cell homeostasis and insulin resistance^24,25^, this work focuses on mitophagy.

Our group has previously described the potential role of the reintroduction of Tsc2 in the Tsc2−/− cells in the control of mitophagy by the up-regulation of one of the essential components in this process, called PTEN-induced kinase 1 (PINK1)^26^. Considering TSC2 as one of the main regulators of mTORC1 activity and autophagy mechanisms, we have elucidated that enhancing TSC2 activity through its deacetylation status promotes a healthy balance between mitochondrial fusion and fission and mitochondrial turnover in order to get a healthy pool of mitochondria that contributes to a better glycemic profile^27,28^.

By using resveratrol, a natural polyphenol with antioxidant effects, our group has demonstrated its effects on the deacetylation of TSC2 protein through a SIRT1-dependant mechanism^18^. In this paper, we have uncovered a key role of TSC2 acetylation in the capacity for its translocation to the lysosomal membrane. In addition, we propose that the deacetylation status of the cell is essential in the capacity to stimulate mitophagy in pancreatic β cells.

## Materials and methods

### Antibodies and reagents

The following antibodies were obtained from Cell Signaling Technology (Beverly, MA): anti-LC3B (#4108), anti-acetyl lysine (#9441), anti-p70S6K (#9202), anti-phospho-p70S6K (Thr389) (#9205), anti-TSC2 (D93F12, #4308), anti-cleaved caspase-3 (#9661), anti-phospho-ACC (Ser 79) (#3661), anti-LAMP1 (#15665), anti-Rheb (#13879), anti-PINK1 (#6946) and anti-phospho-Drp1 (Ser 616) (#3455). Anti-LAMP2A (#ab13524), anti-parkin (#ab77924), anti-TOMM20 (#ab56783) and anti-HADHA (#ab54477) antibodies were obtained from Abcam. Anti-PGC1α (ST1202) was obtained from Calbiochem, anti-SIRT1 (PA5-23063) from Invitrogen and anti-p62 (SQSTM1) (GP62-C-WBC) was obtained from ProGen. Anti-Opa1 (#612606) and anti-Drp1 (#611112) were from BD Biosciences. From Thermo Fisher Scientific were the following secondary antibodies utilized in immunofluorescences: donkey anti-rabbit IgG (H + L) Alexa Fluor 488 (A21206) and donkey anti-mouse IgG (H + L) Alexa Fluor 594 (A21203). From Sigma-Aldrich: anti-β-actin (A5316), anti-tubulin (T6199). Chloroquine (C6628), nicotinamide (N72340), acetyl coenzyme A (A2056), 4′,6-diamidino-2-phenylindole dihydrochloride (DAPI) (D9542) and resveratrol (R5010) were obtained from Sigma-Aldrich. CCCP compound was obtained from Wako Chemicals.

### Cell culture

Mouse insulinoma MIN6 cells were provided by Jun-ichi Miyazaki (Osaka University, Japan) obtained cultured in 15% FBS DMEM high glucose (4,5 g/L), 2 mM l-Glutamine and supplemented with penicillin (12 μg/mL), streptomycin (10 μg/mL), amphotericin B (0,25 μg/mL) and with 50 µM 2-mercaptoethanol. MEF Tsc2+/+ and −/− were generously provided by Dr. Kwiatkowski (Harvard Medical School, Boston). Primary cultures of MEF Sirt1+/+ and Sirt1−/− MEFs were generous gifts of the Maria Monsalve Lab (IIB, CSIC), and were immortalized by retrovirus-mediated transfection of attenuated SV40 T-antigen. After 5 h, the medium was refreshed and 72 h later, cells were selected with puromycin (1 μg/mL) for three weeks. Alternatively, we used immortalized Sirt1+/+ and Sirt1−/− MEFs generously provided by Leonard Guarente (MIT, Boston). All the MEF cell lines were cultured in 10% FBS DMEM high glucose (4,5 g/L), 2 mM l-Glutamine and supplemented with penicillin (12 μg/mL), streptomycin (10 μg/mL) and amphotericin B (0,25 μg/mL). For most of the experiments, cells were seeded and the next day, cells were changed with fresh complete medium for 2 h, after which they were stimulated with either resveratrol 50 µM or nicotinamide 5 mM for 4 h. In some experiments, we cotreated the cells with CQ and with the corresponding stimuli for 24 h.

### Retrovirus production for cell infection

MIN6 cells were co-transfected using lipofectamine 2000 with the lentiviral packaging plasmids pMD2.G (Addgene 12259) and psPAX2 (Addgene, 12260) with pLKO-1-Scrambled-neo or pLKO.1-Tsc2-neo (Addgene, 24150). The supernatants containing lentiviral particles from 48 h after HEK293T transfection were collected and passed through 0.45 μm filters for the elimination of detached cells. MIN6 cells were infected with lentiviral particles in polybrene (8 μg/mL) supplemented media. After 24 h cells were used for experiments.

### Western blotting

After the different treatments, cells were washed twice with PBS and lysed for protein extraction according to standard procedures. Protein concentration determination was achieved by the Bradford dye method, using the Bio-Rad (Hercules, CA) reagent and BSA as standard. Equal amounts of protein (15–20 µg) were submitted to electrophoresis and after SDS–PAGE gels were transferred to Immobilon P PVDF membranes (Merck–Millipore). Then, membranes were blocked with 5% non-fat dried milk and incubated overnight with antibodies at 4 °C. The corresponding bands were visualized using the ECL Western blotting protocol (GE Healthcare, Little Chalfont, UK).

### Immunofluorescence and co-localization analysis

Cells were grown on glass coverslips and fixed using paraformaldehyde 4% for 15 min, permeabilized in PBS with 0.5% Triton X-100 for 10 min and then blocked with (3% BSA, 0,1% Tween 20 in PBS) for 1 h. Cells were incubated o/n at 4 °C with primary antibodies in a wet chamber (1:100 in blocking solution). After this incubation, coverslips were incubated with the corresponding secondary antibodies, at a dilution of 1:100 for 1 h. The microscope was Olympus FV1200 Confocal System (Olympus IX83 inverted microscope), the objective lens was UPLSAPO60XO NA:1.35 and the pinhole was in Auto (optimized for wavelengths and objective lens). The software for image acquisition and processing software was OLYMPUS FLUOVIEW 10-ASW Ver.4.2. The excitation lasers were; for DAPI (405 nm) for Alexa Fluor 488 488 nm) and for Alexa Fluor 594 (594 nm). The spectral detectors were: for DAPI (410–440 nm), for Alexa Fluor 488 (500–530 nm) and for Alexa Fluor 594 (600–650 nm). For co-localization analysis, images were processed with Coloc2 (http://fiji.sc/Fiji). The threshold was obtained automatically using Coste's automatic threshold and the thresholded Manders´ coefficient M1 was determined.

### Electron microscopy

MIN6 Scr and MIN6 TSC2 shRNA were treated with resveratrol and cell extracts were collected to examine mitochondrial structure and autophagosomes. Samples obtained were fixed in 4% paraformaldehyde (Electron Microscopy Tech), and 2.5% glutaraldehyde (Sigma-Aldrich) in 0.1 M sodium phosphate buffer (pH 7.3) for 4 h at 4 °C. Later, samples were postfixed in 1% OsO_4_ (Electron Microscopy Sciences) for 1 h, dehydrated with acetone and embedded in Epon-812 epoxy resin (Taab). Thin sections (60–70 nm) were obtained with an Ultracut E (Leica) ultramicrotome, stained with lead citrate and observed under a JEM-1010 transmission electron microscope (JEOL) in the Electron Microscopy Center at Complutense University of Madrid (Madrid, Spain). For the quantification of the number of mitochondria per image and the mitochondrial length in microns we used the free software image J. For the quantification of the number of mitochondria, we counted multiple images and we related all the values to the control condition of MIN6 Scr. For the analysis of the mitochondrial length, we used several images with different amplifications and, after its calibration, we draw a line along the major axis of the mitochondria and the program indicated the length in microns.

### Immunoprecipitation (IP)

After the stimulation of the cells with the corresponding treatments, the cells were washed with PBS, cells were lysed and the corresponding protein concentrations were determined using the Bradford method, as previously explained in the western blotting section. Then, for the IP in pancreatic β cells and from MEF cell lines, we used 1 mg of the total amount of protein and the corresponding antibody (PGC1-α, in the case of pancreatic β cells MIN6 Scr and MIN6 TSC2 shRNA or TSC2, in the case of the different MEF cell lines and in MIN6 Scr pancreatic β cells) at 2 μg of antibody/500 μg of protein for 24 h at 4 °C under gentle agitation by rotation. The next day, we added 30 µL of slurry protein-G-agarose at 50% to the tubes and incubated for 1 h at 4 °C under gentle agitation by rotation. Then, after centrifugation at 1000 × *g* at 4 °C for 15 s to obtain the immune complexes, the supernatants were discarded and 500 µL of PBS was added for the washing step and repeated two times. After the washing step, the beads were resuspended in 30 µL of sample buffer 2 × with 2% of β-mercaptoethanol and heated at 98 °C for 3 min. After that, the supernatants were loaded in SDS-PAGE gels and continued as previously explained in the western blotting section.

### Statistical analysis

Statistically significant differences between mean values were determined using the unpaired Student’s *t*-test (when we compared two specific groups) or one-way-ANOVA test (when we compare more than 2 groups) in the GraphPad statistical analysis software package. Differences were considered statistically significant at p ≤ 0.05 (*^/#^p ≤ 0.05; **^/##^p ≤ 0.01; ***p ≤ 0.005).

## Results

### The translocation of TSC2 to the lysosome is dependent on its acetylation status.

**Figure 1:**
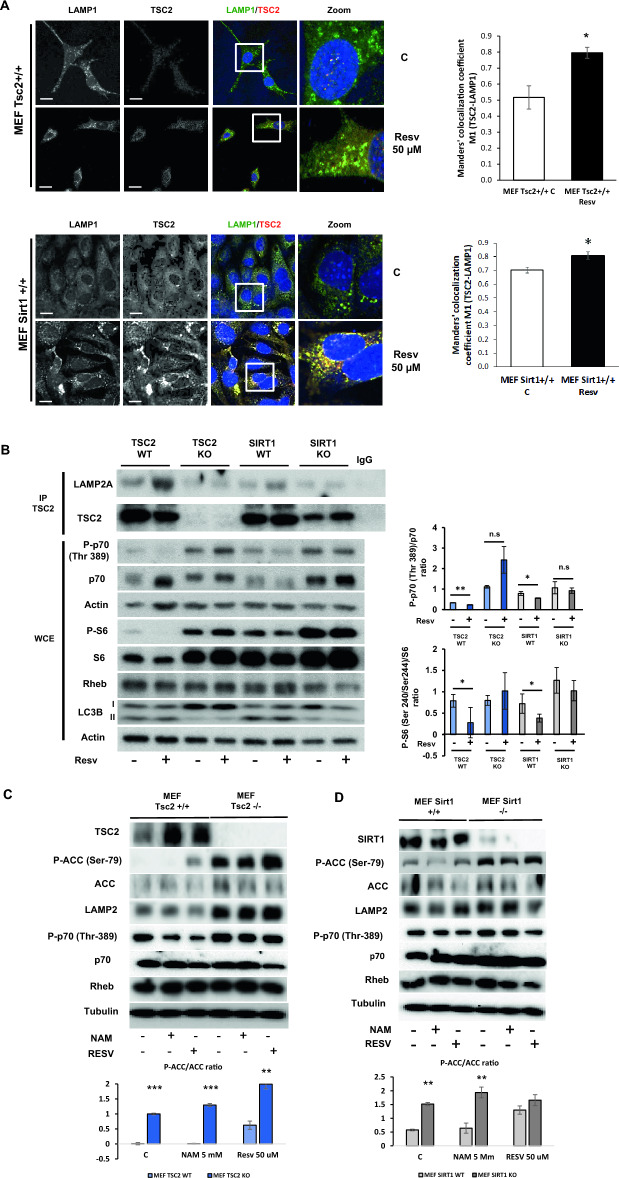
TSC2 localizes in the lysosomal membrane when it is deacetylated in MEFs. (**A**) Immunofluorescence showing the association between LAMP1 (red channel) and TSC2 (green channel) in MEF TSC2+/+ and Sirt1+/+ cells under control conditions and after resveratrol treatment at 50 µM. The graphs represent the Manders’ colocalization coefficient M1 of TSC2-LAMP1 in either MEF Tsc2+/+ and MEF Sirt1+/+. The values represent means and SD; n = 3 independent experiments. *p ≤ 0.05 comparing MEF Tsc2+/+ control versus resveratrol treatment and MEF Sirt1+/+ control and after the addition of resveratrol. Bars, 20 μm. (**B**) Immunoprecipitation of TSC2 and western blotting against LAMP2A under normal conditions and after the treatment with resveratrol at 50 µM in either MEF Tsc2+/+, Tsc2−/−, Sirt1+/+ and Sirt1−/−. It is shown in the last lane a negative control using a non-specific antibody for the immunoprecipitation. It is also shown in the whole cell extracts (WCE) the mTORC1/p70S6K signalling pathway with the corresponding quantification and statistical analysis. (**C**,**D**) Representative western blots corresponding to the mTORC1 and AMPK signalling pathways using either nicotinamide (5 mM), resveratrol (50 µM) or control conditions either comparing MEF Tsc2+/+ and −/− cells (**C**) or MEF Sirt1+/+ and −/− cells in (**D**). It is shown in the graphs (in **C** and in **D**) the densitometry corresponding to the P-ACC/ACC ratio in all the cell lines analyzed. The values represent means and SD; n = 6. **p ≤ 0.01, ***p ≤ 0.001 MEF Tsc2+/+ and Sirt1+/+ vs MEF Tsc2−/− and Sirt1−/−.

We have previously demonstrated that sirtuin-1 (Sirt1) modulation, by using the inhibitor nicotinamide (NAM) or the inducer resveratrol (RESV), regulates TSC2 acetylation levels and, concomitantly, mTORC1 signalling in both mouse embryo fibroblasts (MEF) and pancreatic β cells^18^. However, the effect of TSC2 acetylation in its capacity to translocate to the lysosomal membrane is unknown. Using MEF TSC2+/+ cells, we observed that in response to RESV, there was an increase in the colocalization signal of TSC2 with the lysosomal marker LAMP1, suggesting that TSC2 is recruited to the lysosome in its deacetylated form. As negative control of TSC2 immunofluorescence, we used MEF Tsc2−/− cells (Supplementary Fig. 1A). For corroborating the role of acetylation in the capacity of TSC2 to translocate to the lysosome, using MEF Sirt1+/+ cells treated with RESV. Then, we observed a similar colocalization signal to what we observed in MEF Tsc2+/+ cells (Fig. 1A). We previously determined that in Sirt1−/− cells there was a hyperacetylation and concomitant ubiquitination and degradation of TSC2 protein. These changes were associated with an increase in mTORC1 activity^18^. In order to determine the effect of TSC2 acetylation status with a correct recruitment to the lysosome, we analyzed TSC2-LAMP2A interaction by immunoprecipitation in all the cell lines (MEF Tsc2+/+, Tsc2−/−, MEF Sirt1+/+ and Sirt1−/−) either under basal conditions or in response to RESV. In MEF Tsc2+/+ and Sirt1+/+ cells there was a clear association between TSC2 and LAMP2A after the treatment with resveratrol. Very importantly, in Sirt1−/− cells there was a clear impairment in the capacity of TSC2 to translocate to the lysosomal membrane using western blotting analysis (Fig. 1B) as well as using immunofluorescence (Supplementary Fig. 1B). Interestingly, in MEF Tsc2−/−, apart from the expected hyperactivation of mTORC1 signalling, we observed a paradoxical hyperactivation of AMPK signalling pathway at the same time, which suggests a metabolic conflict in these cells (Fig. 1C). These unexpected hyperactivation of both mTORC1 and AMPK signalling pathways were also observed in MEF Sirt1−/− cells (Fig. 1D).

### The acetylation status activates mTORC1 signalling pathway in MIN6 pancreatic β cells

**Figure 2:**
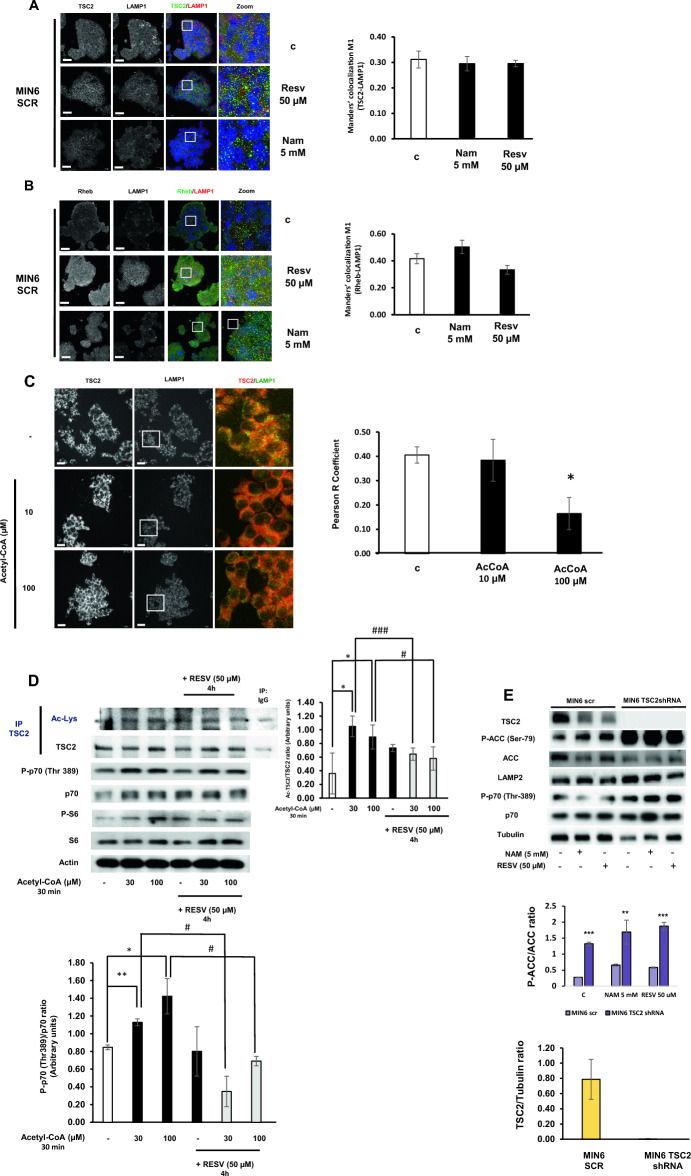
Acetyl CoA disrupts the interaction between TSC2 and lysosome in MIN6 pancreatic beta cells. (**A**) Immunofluorescence analyzing the colocalization signal between TSC2 and LAMP1 proteins in MIN6 Scr cells. The graphs represent the Manders’ colocalization coefficient M1 of TSC2-LAMP1 after NAM or RESV treatments. The values represent means and SD; n = 4 independent experiments. Bars, 20 μm. (**B**) Immunofluorescence analyzing the colocalization signal between Rheb and LAMP1 proteins in MIN6 Scr cells. The graphs represent the Manders’ colocalization coefficient M1 of Rheb-LAMP1 after NAM or RESV treatments. The values represent means and SD; n = 4 independent experiments. Bars, 20 μm. (**C**) Immunofluorescence studying the colocalization signal between TSC2 and LAMP1 in MIN6 Scr cells after the stimulation with acetyl-CoA at either 10 or 100 µM. The values represent means and SD; n = 3 independent experiments. One-way ANOVA was performed with Tukey's multiple comparisons test as post hoc. *p ≤ 0.05 comparing control versus AcCoA 100 µM. Bars, 20 μm. (**D**) Representative western blots showing the acetylation status of TSC2 protein in response to increasing concentration of Acetyl-CoA and its reversion with resveratrol. The graph represents the densitometric analysis of Ac-TSC2/TSC2 and phospho-p70 (Thr 389)/p70 ratios. *p ≤ 0.05 and **p ≤ 0.01 comparing control versus either AcCoA 30 µM and AcCoA 100 µM. ^#^p ≤ 0.05 and ^###^p < 0.001 comparing AcCoA at either 30 µM or 100 µM in the absence of resveratrol with the presence of resveratrol. (**E**) Representative western blots corresponding to the mTORC1 and AMPK signalling pathways using either nicotinamide (5 mM), resveratrol (50 µM) or control conditions in MIN6 Scr cells and MIN6 Tsc2 shRNA cells. The graph represents the densitometric analysis of the P-ACC/ACC ratio comparing both cell lines. The values represent mean and SD; n = 6. **p < 0.01, ***p ≤ 0.001 MEF Tsc2+/+ and Sirt1+/+ vs MEF Tsc2−/− and Sirt1−/−. **p ≤ 0.01, ***p ≤ 0.001 MIN6 Tsc2 Scr vs MIN6 Tsc2 shRNA.

Pancreatic β cells are the key regulators of energy homeostasis in the organism through the correct secretion of insulin, which assures energy supply to the cells, in the form of either amino acids or glucose, for the synthesis of macromolecules favouring cell growth and inducing mTORC1. Since, in these cells is necessary a correct coordination between the presence of nutrients with insulin secretion, we wanted to determine whether or not TSC2 is recruited to the lysosome for mTORC1 activity regulation. Unexpectedly, we did not observe any change in TSC2 translocation to the lysosome in response to RESV (Fig. 2A). Then, we analyzed Rheb protein, which is the target of TSC2 and it is essential for mTORC1 induction. Firstly, we determined that, indeed, Rheb protein is located in the lysosomal membrane under basal conditions. Using nicotinamide (NAM), as an allosteric inhibitor of sirtuins activity, we observed a mild recruitment of TSC2 to the lysosome. In contrast, in response to RESV there was a tendency to diminish this interaction. These changes were not statistically significant (Fig. 2B). In order to clearly determine the effect of acetylation on the capacity of TSC2 to translocate to the lysosomes, we stimulated MIN6 Scr cells with acetyl-CoA as the main acetyl group donor in the cell. The addition of acetyl-CoA, in a dose-dependent manner, was sufficient to disrupt the interaction between TSC2 and the lysosomal marker LAMP1 in MIN6 Scr cells (Fig. 2C). These data suggest that the deacetylated form of TSC2 is essential for its translocation to the lysosomal surface and that, acetyl-CoA is able to disrupt the interaction between TSC2 and LAMP1 in these cells. Importantly, the addition of acetyl-CoA was related to an increase in mTORC1 signalling pathway, which was abrogated by the pretreatment with resveratrol (Supplementary Fig. 2). Very importantly, when we analyzed the effect of acetyl-CoA on TSC2 acetylation, we observed an induction of the acetylation status of TSC2 in response to acetyl-CoA. In contrast, when we pre-treated the cells with resveratrol, the acetylation status of TSC2 was almost reverted to basal conditions. Concomitantly, there was a reduction in mTORC1 induction (Fig. 2D). In addition, in MIN6 Tsc2 shRNA there was a similar hyperactivation in both mTORC1 and AMPK signalling pathways in a similar way as it was previously observed in MEF Tsc2−/−, suggesting an analogous metabolic conflict in pancreatic β cells after the deletion of TSC2 (Fig. 2E).

### Resveratrol facilitates mitophagy in MIN6 Scr pancreatic β cells

**Figure 3:**
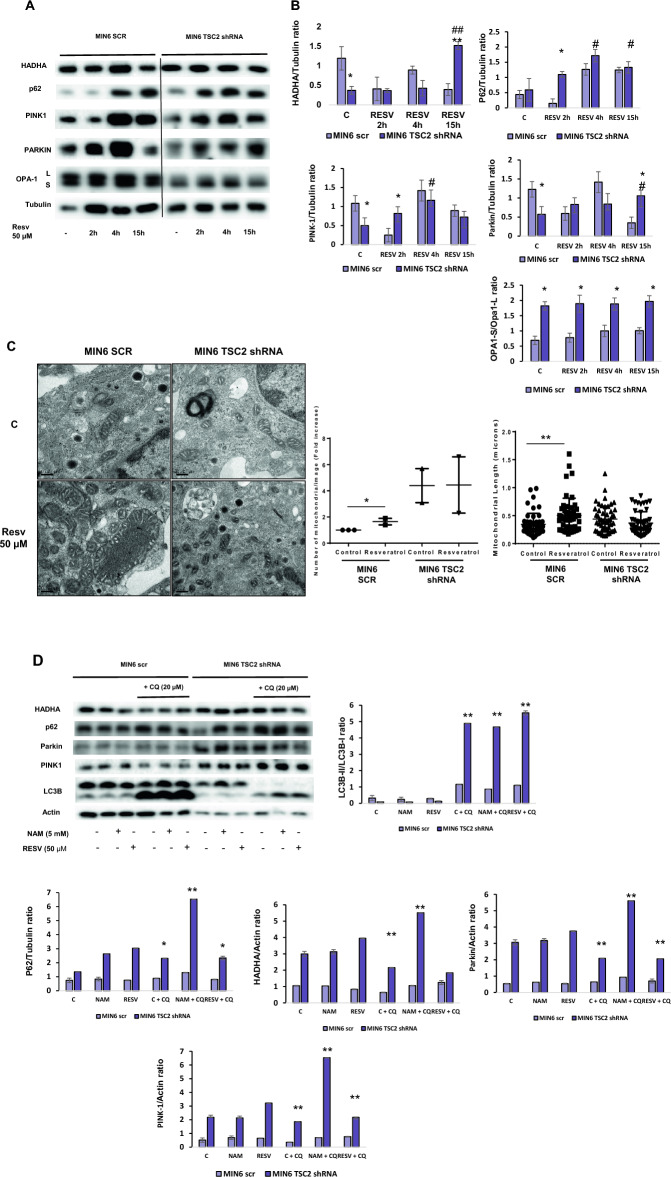
Resveratrol induces both autophagic and mitophagic fluxes in MIN6 Scr pancreatic beta cells. (**A**) Representative western blots of different mitophagy markers in MIN6 Scr and MIN6 Tsc2 shRNA pancreatic beta cells treated or not with resveratrol at 50 µM using a time-course analysis. (**B**) The graphs represent the densitometric study showing the mean and SD; n = 4 independent experiments. One-way ANOVA was performed with Tukey's multiple comparisons test as post hoc. *p ≤ 0.05, **p ≤ 0.01 MIN6 Tsc2 Scr vs MIN6 Tsc2 shRNA; ^#^p ≤ 0.05 MIN6 treated with resveratrol 50 µM vs control. (**C**) Transmission electronic microscopy (TEM) images obtained from MIN6 Tsc2 Scr and Tsc2 shRNA treated or not with resveratrol 50 µM. The bar represents 0.2 µm. *p ≤ 0.05 and **p ≤ 0.01 comparing control versus resveratrol in the MIN6 Scr cells. (**D**) Representative western blots for the study of autophagic and mitophagic fluxes in MIN6 Scr and Tsc2 shRNA pancreatic beta cells treated with either nicotinamide (5 mM) or resveratrol (50 µM) in the presence or in the absence of a pre-treatment with chloroquine (CQ) for 24 h. The graphs correspond to the densitometric analysis of all the blots showing the mean ± SD, n = 3. One-way ANOVA was performed with Tukey's multiple comparisons test as post hoc. **p ≤ 0.01, ***p ≤ 0.001 MIN6 Tsc2 Scr vs MIN6 Tsc2 shRNA.

Since resveratrol has been involved in the enhancement of mitochondrial function, protecting from metabolic decline during aging and different diseases^29^, we analyzed whether resveratrol could facilitate mitochondrial recycling through mitophagy induction in MIN6 pancreatic β cells. First, we performed a time course of resveratrol in MIN6 Scr cells in comparison with MIN6 Tsc2 shRNA. We observed an increase in the fissioned status of the mitochondria in MIN6 TSC2 shRNA cells, because of the higher Opa-S/Opa-L ratio found under basal levels and in response to resveratrol compared with MIN6 Scr cells. In MIN6 Scr cells there was an increase in PINK-1, Parkin and HADHA proteins, which are either two proteins involved in one of the main mitophagic pathways (PINK1 and Parkin) or a matrix protein, which is eliminated by mitophagy (HADHA). In all the proteins, there was a maximum protein level at 4 h in these cells. After this time point of 4 h, there was a decrease in all of these proteins, suggesting that there is an active mitophagic process through PINK-1/Parkin mitophagic pathway. In contrast, in MIN6 Tsc2 shRNA, although there was a similar increase in PINK1, Parkin and HADHA proteins, the signal of Parkin protein was stable beyond 15 h in these cells, suggesting an accumulation of labelled mitochondria which were not correctly degraded via mitophagy (Fig. 3A,B). In addition, using electron microscopy, we detected an increase in both the number of mitochondria as well as the mitochondrial length, after the stimulation with resveratrol. Collectively, these data potentially indicates that resveratrol, apart from inducing mitophagy (Fig. 2A,B), can stimulate mitochondrial biogenesis at the same time (Fig. 3C), favouring a correct mitochondrial turnover. We decided to analyze the autophagic and mitophagic flux by using chloroquine (CQ). We observed that in MIN6 Scr cells, in response to resveratrol, there was a reduction in mitochondrial mass using HADHA protein levels and, after CQ treatment, an accumulation in both LC3B and HADHA occurred, suggesting positive autophagic and mitophagic fluxes, respectively. In contrast, MIN6 Tsc2 shRNA has an expansion in mitochondrial mass represented in HADHA protein expression levels and, more importantly, neither decrease in response to resveratrol nor did not increase after CQ treatment, which reflects an impairment in mitochondrial flux in this cell line (Fig. 3D).

The accumulation of p62, PINK1 and Parkin protein levels at the basal state in MIN6 Tsc2 shRNA, corroborated the alteration in mitochondrial degradation that it was previously indicated. In addition, there was an increase in the accumulation of PINK1 protein levels but not of Parkin protein levels in response to CQ, suggesting an accumulation of labelled mitochondria that could not be correctly degraded by mitophagy. All these data indicate a defective mitophagic flux in these cells compared with MIN6 Scr cells (Fig. 3D). In order to corroborate this alteration in MIN6 Tsc2 shRNA, we performed an experiment using CCCP as a mitochondrial uncoupler and a positive control of mitophagy. The experiment with this agent confirmed that mitochondria in MIN6 TSC2 shRNA cells are constitutively fissioned and delivered into mitophagy (Supplementary Fig. 3).

### Resveratrol induces mitochondrial improvement by enhancing mitophagy and mitochondrial biogenesis in MIN6 Scr pancreatic β cells

**Figure 4:**
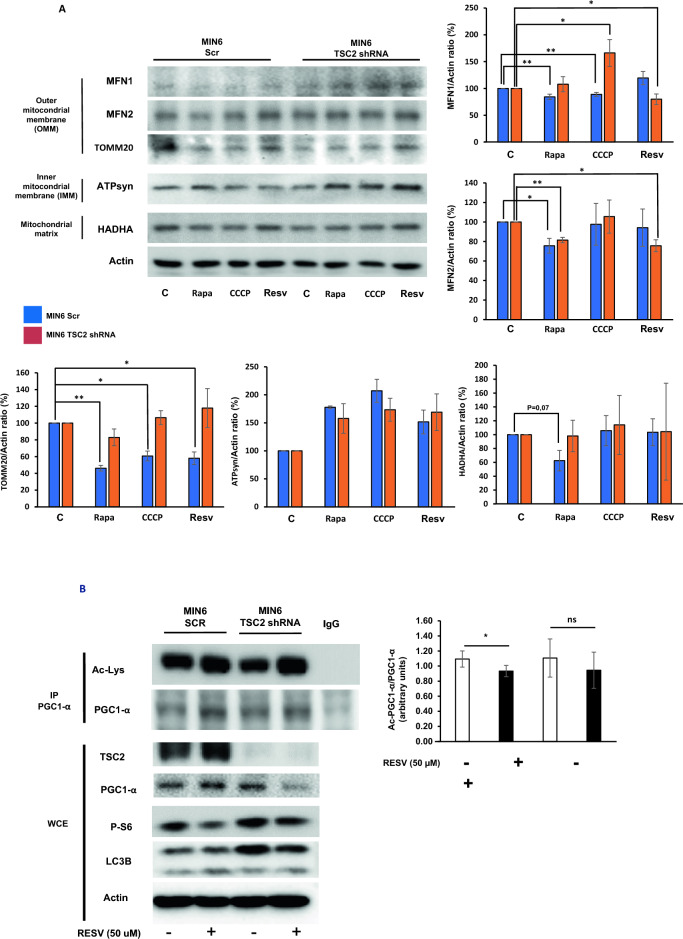
Resveratrol induces mitophagy and mitochondrial biogenesis by regulating PGC1-α acetylation in MIN6 Scr pancreatic beta cells. (**A**) Western blotting analysis showing the study of different proteins from the different mitochondrial compartments for study mitophagy at 24 h in response to either rapamycin, CCCP or resveratrol. The graphs correspond to the quantification analysis of the protein changes in response to the different stimuli expressed in percentage (n = 3). (**B**) Immunoprecipitation analysis of PGC1-α for analyzing the acetylation status of the protein in response or not to resveratrol for 4 h in MIN6 Scr and Tsc2 shRNA cells. The graph corresponds to the quantification analysis showing the fold increase in the ratio of Ac-PGC1-α/PGC1-α (n = 3).

After characterizing the autophagic and mitophagic fluxes in these cells, we wanted to analyze how resveratrol could improve mitophagy in long term. With this purpose, we stimulated MIN6 Scr and Tsc2 shRNA cells with resveratrol and with two other positive controls of autophagy, rapamycin and CCCP. In MIN6 Scr cells, there was a reduction in TOMM20 protein levels in response to all the treatments in MIN6 Scr cells. In contrast, in MIN6 Tsc2 shRNA, there was an accumulation of TOMM20 protein, indicating a defect in the elimination of mitochondria. In the rest of the proteins, we only observed a reduction in MFN1 in response to rapamycin and CCCP and, in MFN2 in response to rapamycin, which were abolished in MIN6 Tsc2 shRNA cells (Fig. 4A). Since it has been demonstrated that resveratrol is able to induce mitochondrial biogenesis in different cell lines and models^29^ we decided to analyze the acetylation status of PGC1-α, as one of the master regulators of this process^30^. Our results showed that resveratrol decreased the acetylation levels of PGC1-α in MIN6 Scr. In contrast, in MIN6 TSC2 shRNA, we did not see any change in the acetylated status of PGC1-α in response to resveratrol under these conditions (Fig. 4B), confirming that the acetylation status impairs mitochondrial biogenesis in MIN6 Tsc2 shRNA cells in response to resveratrol.

## Discussion

Pancreatic β cells are extremely sensitive to environmental changes in metabolism and energetic stress. One of the main coordinators under these conditions is mTORC1 signalling pathway. mTORC1 presents a very tight regulation through multiple partners, being TSC2 essential in its activity^20^. It has been proposed that TSC2 inhibits mTORC1 activity by its recruitment to the lysosome under different situations such as energy stress or energy deprivation and in a variety of cell lines^6,31,32^. However, the molecular mechanism by which TSC2 is recruited to this organelle is unknown. In this paper we have uncovered that acetylation level is essential for its recruitment using different cellular systems such as fibroblasts and pancreatic β cells. Very importantly, we have observed that in MEF Sirt1−/−, with a global increase in the acetylation status of the cells^33^, including TSC2^18^, there is a relevant impairment in TSC2 translocation to the lysosomal surface. In addition, we could observe a reduction in the total amount of TSC2 protein levels, which is coherent with the increase in TSC2 ubiquitination found in MEF Sirt1−/− cells^18^. In this regard, in pancreatic β cells, although we did not observe any change in TSC2 recruitment in response to resveratrol, the addition of acetyl-CoA was capable of disrupting TSC2-LAMP1 association and facilitating the activation of the mTORC1 signalling pathway. Moreover, acetyl-CoA increased the acetylation status of TSC2, which was related with an increased in mTORC1 activation and, resveratrol, could revert both the acetylation status of TSC2 and mTORC1 stimulation. These data indicate that acetylation status controls TSC2 recruitment to the lysosomal membrane, controlling mTORC1 activity, in both MEF and pancreatic β cells.

The hyperactivation of both mTORC1 and AMPK signalling pathways at the basal state has been observed in TSC2−/− cells previously^34^. Then, TSC2 deletion can generate a “glucose addiction” phenomenon that is associated with a higher susceptibility to glucose deprivation and dying by apoptosis, indicating that TSC2 is essential in the control of energetic homeostasis^6,34,35^. This paradoxical event indicates a higher basal energy demand, being not possible to maintain a higher proliferation rate and a higher glucose consumption than Tsc2+/+ cells, stimulating AMPK signalling pathway. This effect was also observed in pancreatic β cells when we knocked down TSC2 protein.

Although we did not see basal acetylation differences in PGC1-α and in Tsc2−/− cells, it is known that TSC deficiency could alter the acetylation profile. For instance, TSC2 deletion in neurons, increases microtubule acetylation^36^. In contrast, TSC1 loss leads to a hypoacetylation of the molecular chaperone Hsp90^37^. Although mTORC1 is involved in mitochondrial biogenesis^38^, we did not observe any change in the acetylation status of PGC1-α in MIN6 Tsc2 shRNA in response to resveratrol, suggesting that TSC2 deletion have some detrimental effect in the control of PGC1-α activation. In fact, PGC1-α deacetylation is controlled by Sirt1 activity, being the master regulator of mitochondrial biogenesis^39,40^. And, Sirt1 activity is regulated by nutrients, indicating that glucose can revert the activation of Sirt1^40^, and hence PGC1-α induction. In addition, glucose stimulates mTORC1 signalling and can promote protein acetylation, affecting the catalytic activity of different proteins and cell function^41,42^ being altered in different metabolic diseases including diabetes and, in its complications,^43,44^. In this regard, TSC2 acetylation is related to mTORC1 activation and cell proliferation and impairs the protective role of autophagy mechanisms in cell homeostasis^18^. Resveratrol treatment was able to stimulate the AMPK/SIRT1/PGC-1α axis in pancreatic β cells, which deacetylates TSC2 and improves its inhibitory capacity towards mTORC1. Moreover, resveratrol treatment besides enhancing mitochondrial degradation machinery, facilitated an improvement in mitochondrial biogenesis in pancreatic β cells, favored by PGC-1 α deacetylation.

Previous data indicates that TSC2 elimination impairs mitophagy, showing mitochondrial alterations involved in insulin resistance, obesity and other diseases^26,45^. In the absence of TSC2 protein, resveratrol treatment could induce autophagy through LC3 and FOXO1 deacetylation in a SIRT1-dependent manner^46,47^, which counteracts mTORC1 activity, playing a pivotal role in the maintenance of a healthy pool of mitochondria.

**Figure 5:**
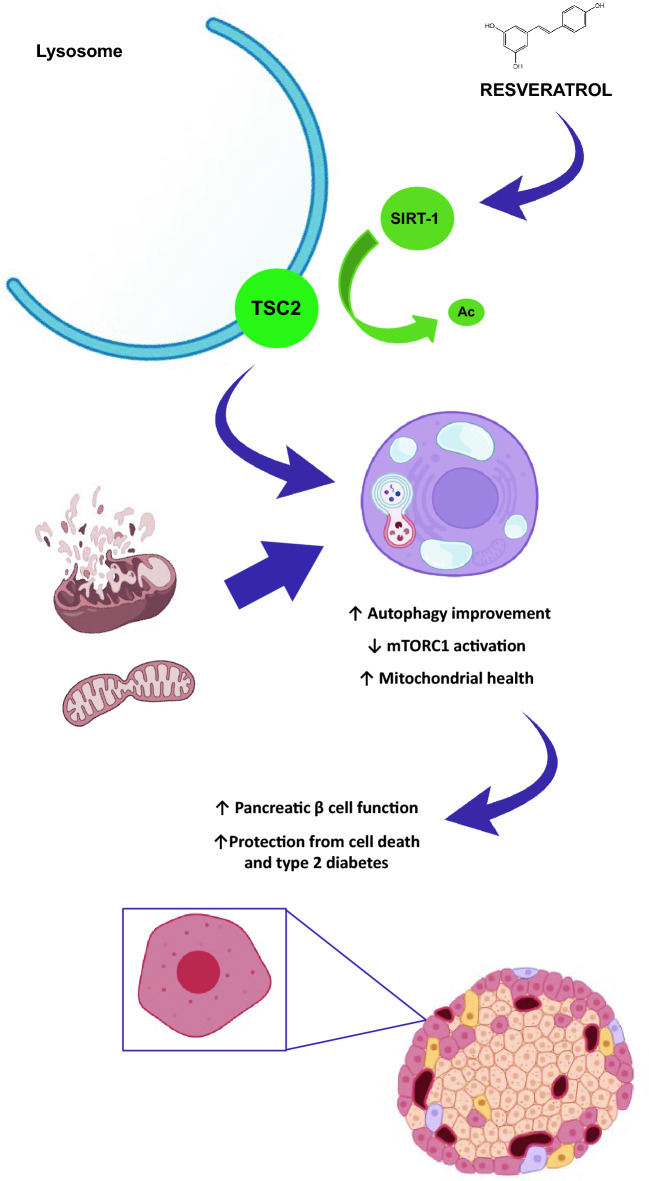
Scheme depicting the most important results obtained in the manuscript indicating the role of resveratrol in the control of acetylation status of TSC2 and its recruitment to the lysosomal membrane. It shows the involvement of resveratrol in the maintenance of a healthy pool of mitochondrial by enhancing mitophagy and mitochondrial biogenesis.

In summary, resveratrol facilitates TSC2 recruitment to the lysosome, inhibiting mTORC1 pathway and improving the mitochondrial turnover, contributing to pancreatic β cell homeostasis in a TSC2 and SIRT-1 dependent manner, which is crucial in avoiding metabolic disorders such as type 2 diabetes. In Fig. 5 it is resumed the most important conclusions derived from the present paper.

### Supplementary Information

Supplementary Information 1.

Supplementary Information 2.

Supplementary Information 3.

## Data Availability

The datasets used and/or analyzed during the current study are available from the corresponding author on reasonable request. All images derived from western blots generated during this study are included in this published article as supplementary information with the name "original blots”.
