# Supplementary material for: Regulation of TSC2 lysosome translocation and mitochondrial turnover by TSC2 acetylation status
Source: Sci Rep. 2024 May 31;14:12521. doi: 10.1038/s41598-024-63525-7 (PMC11143182; doi:10.1038/s41598-024-63525-7)

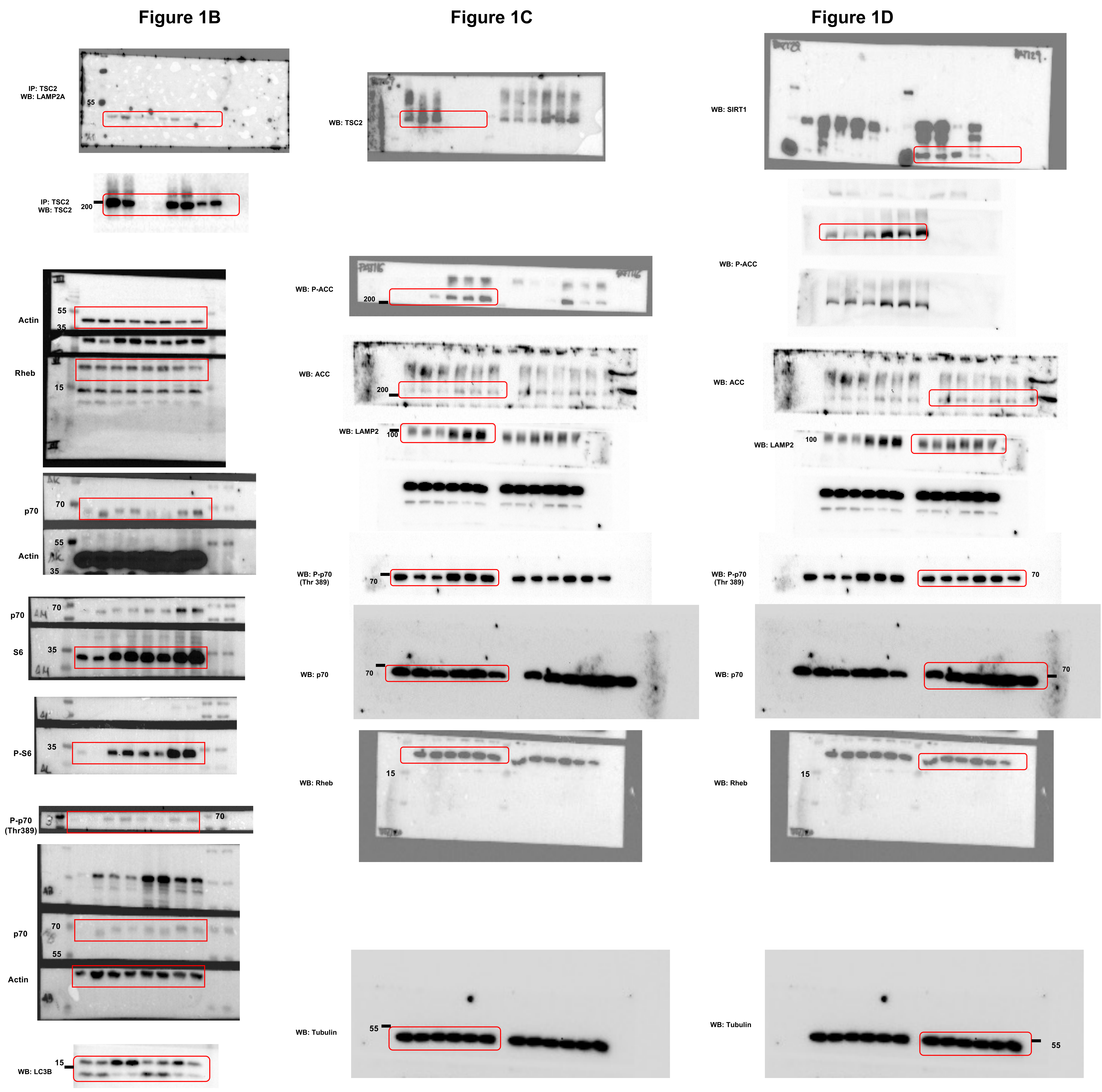
Figure 1B
Figure 1C
Figure 1D
IP: TSC2
WB: LAMP2A
55
IP: TSC2
WB: TSC2
200
Actin
55
35
Rheb
15
p70
70
Actin
55
35
p70
70
S6
35
P-S6
35
P-p70
(Thr389)
70
p70
70
55
Actin
WB: LC3B
15
WB: TSC2
WB: P-ACC
200
WB: ACC
200
WB: LAMP2
100
WB: P-p70
(Thr 389)
70
WB: p70
70
WB: Rheb
15
WB: Tubulin
55
WB: SIRT1
WB: P-ACC
WB: ACC
WB: LAMP2
100
WB: P-p70
(Thr 389)
70
WB: p70
70
WB: Rheb
15
WB: Tubulin
55

Figure 2D

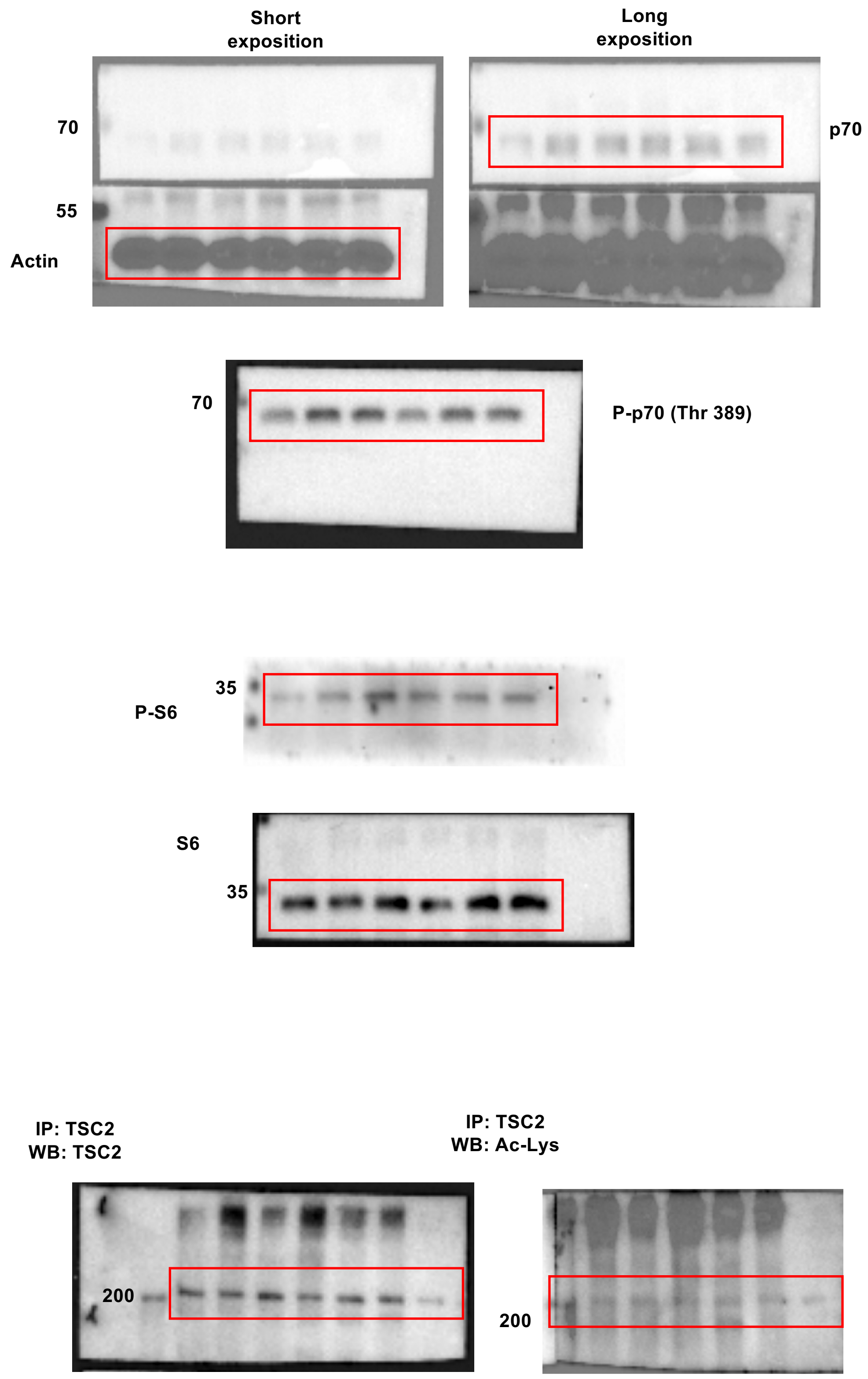

Figure 2E

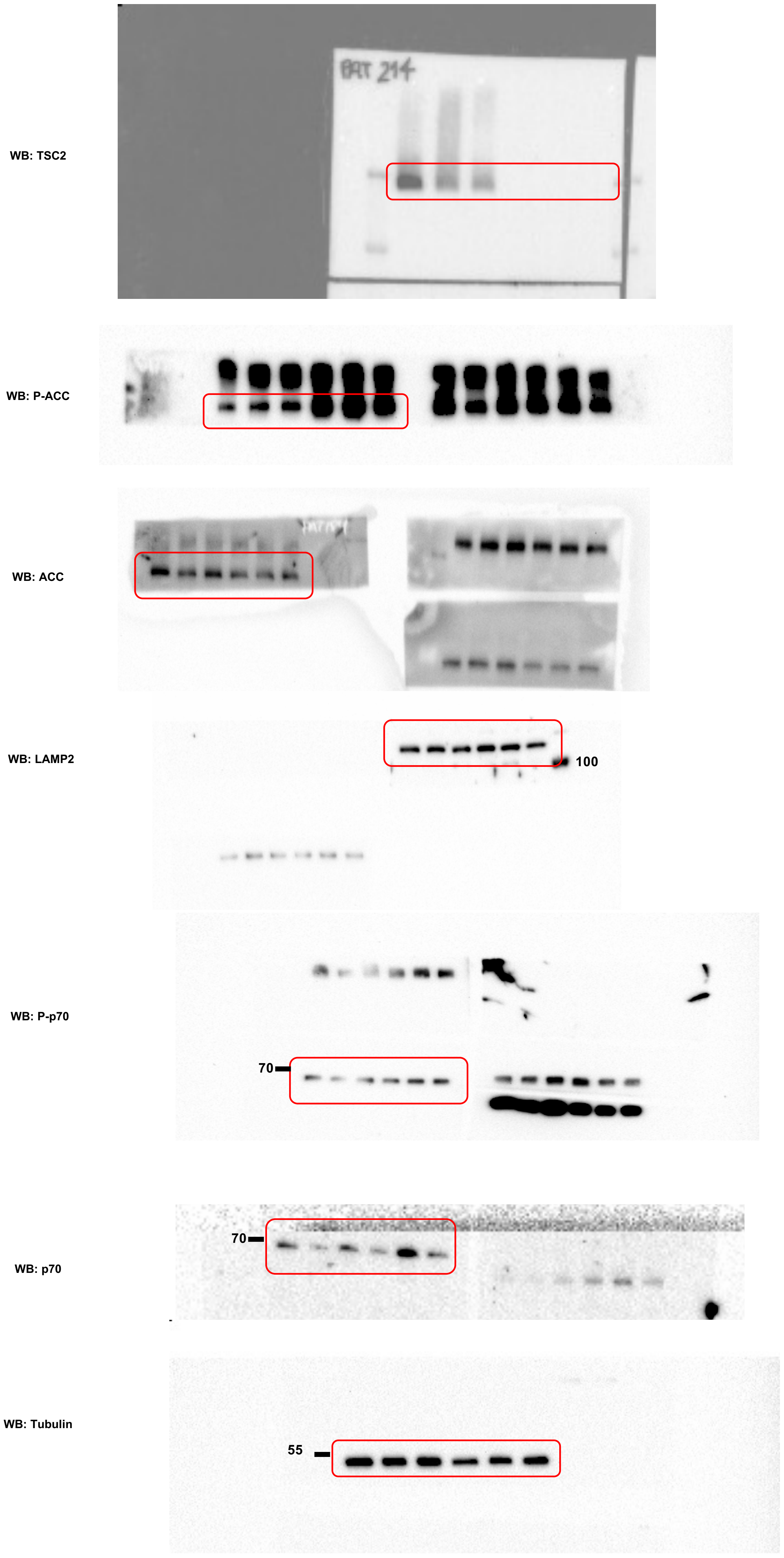

Figure 3A

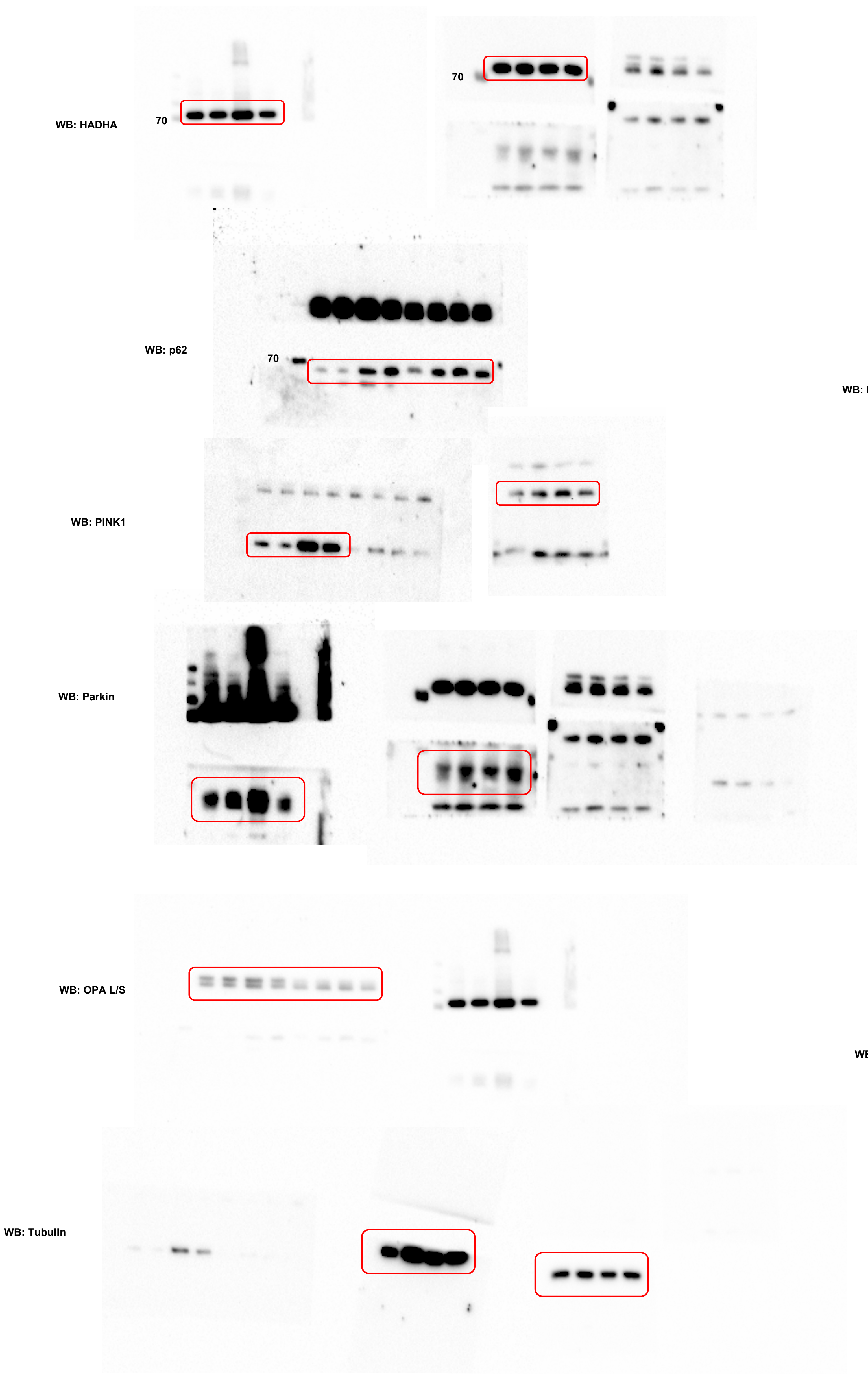

Figure 3D

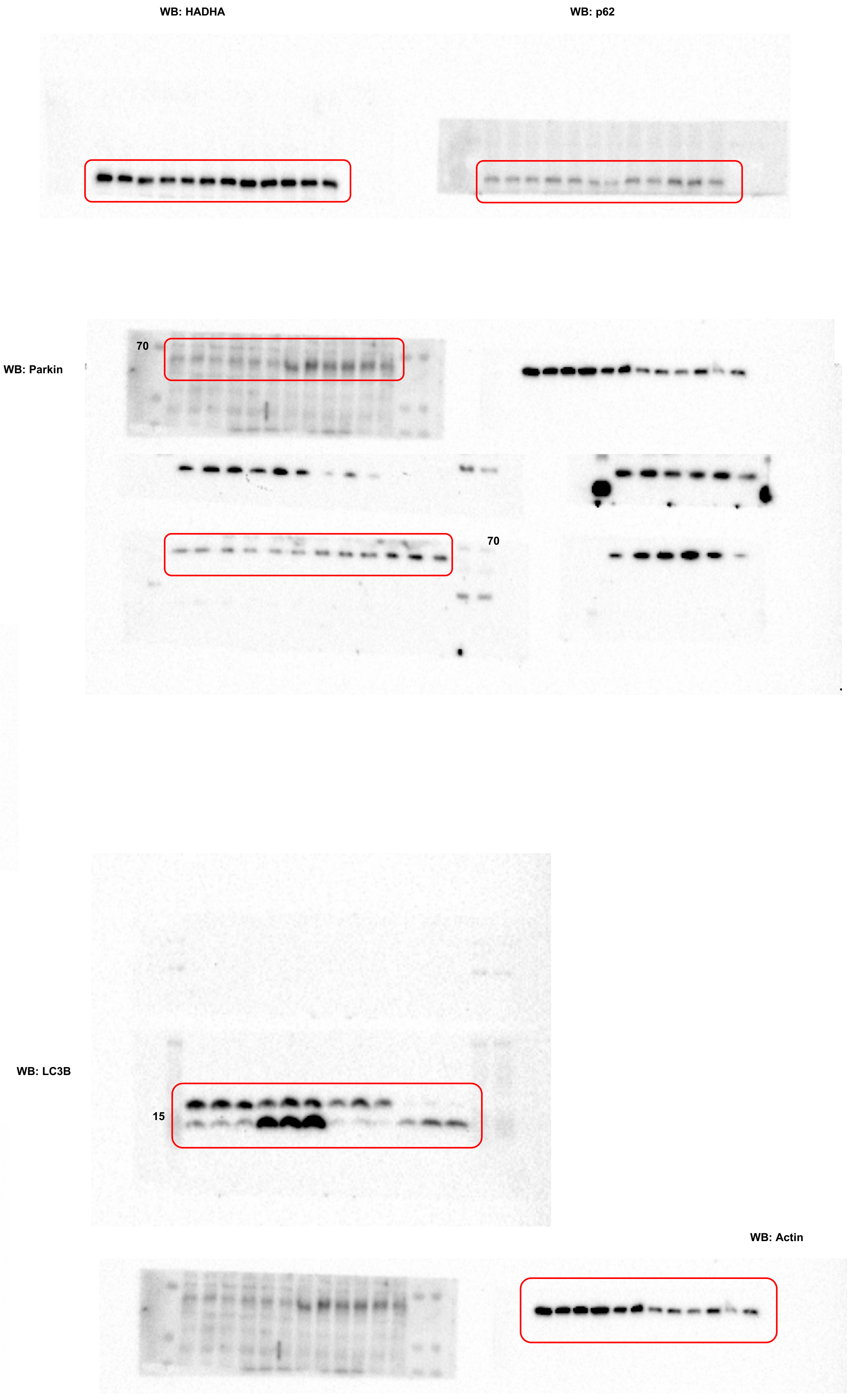

Figure 4A

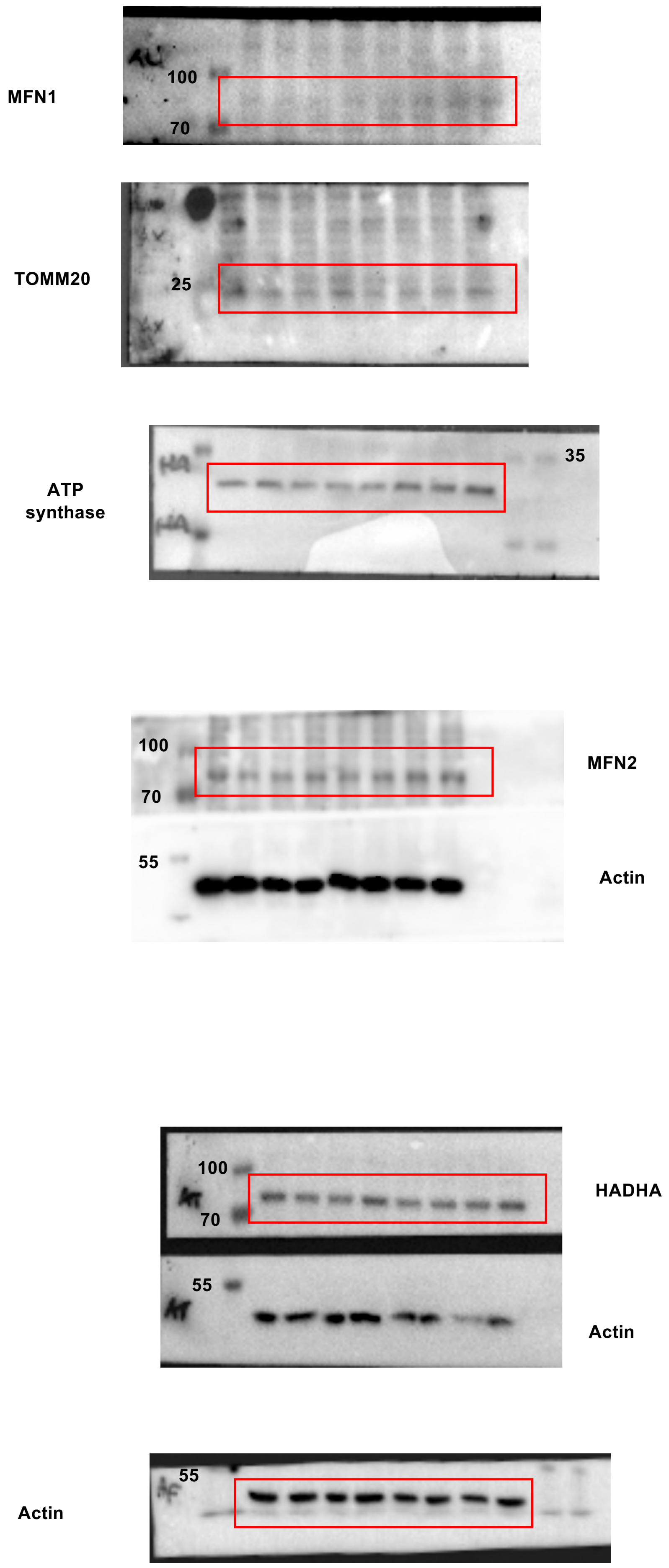

Figure 4B

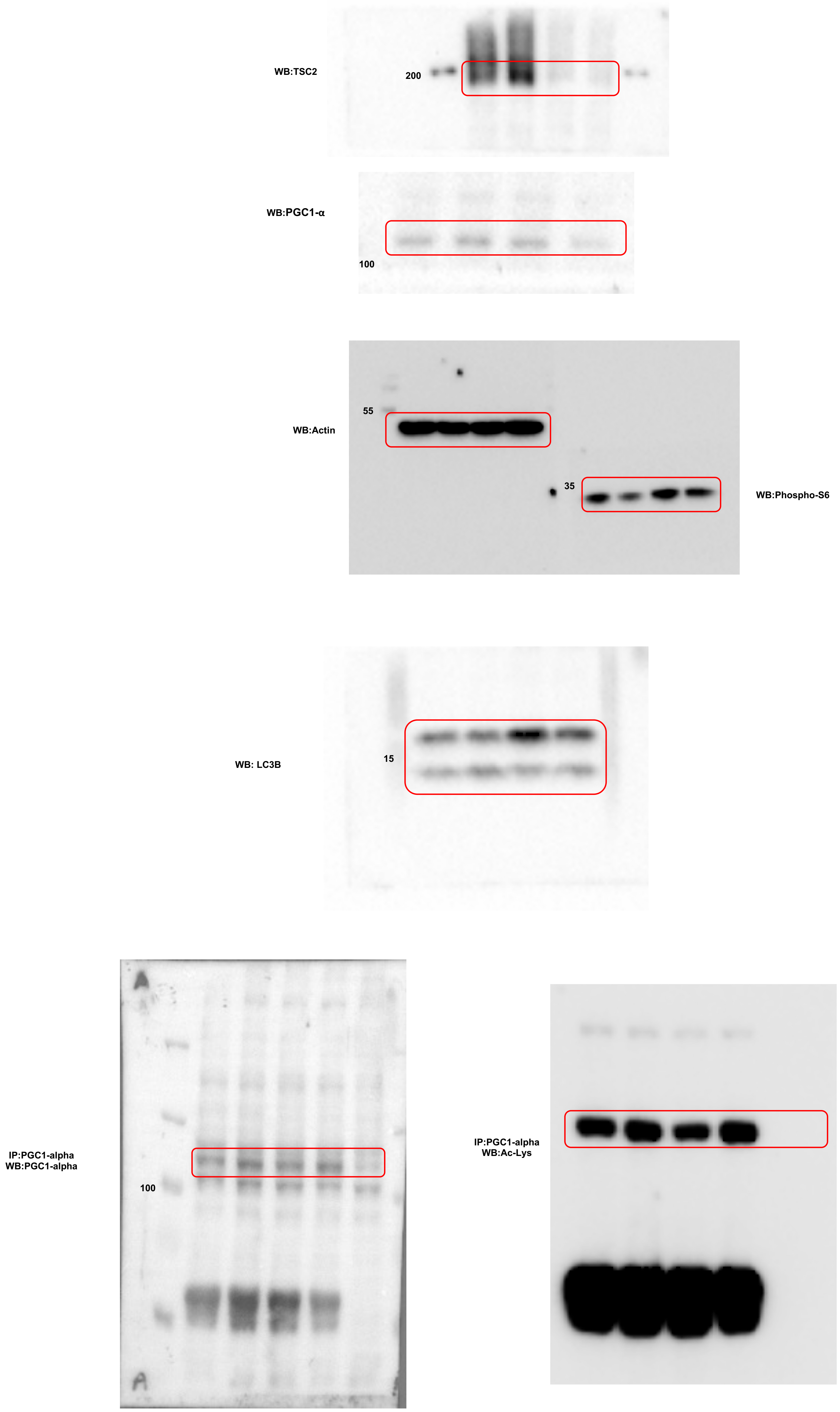

Supplementary Figure 2

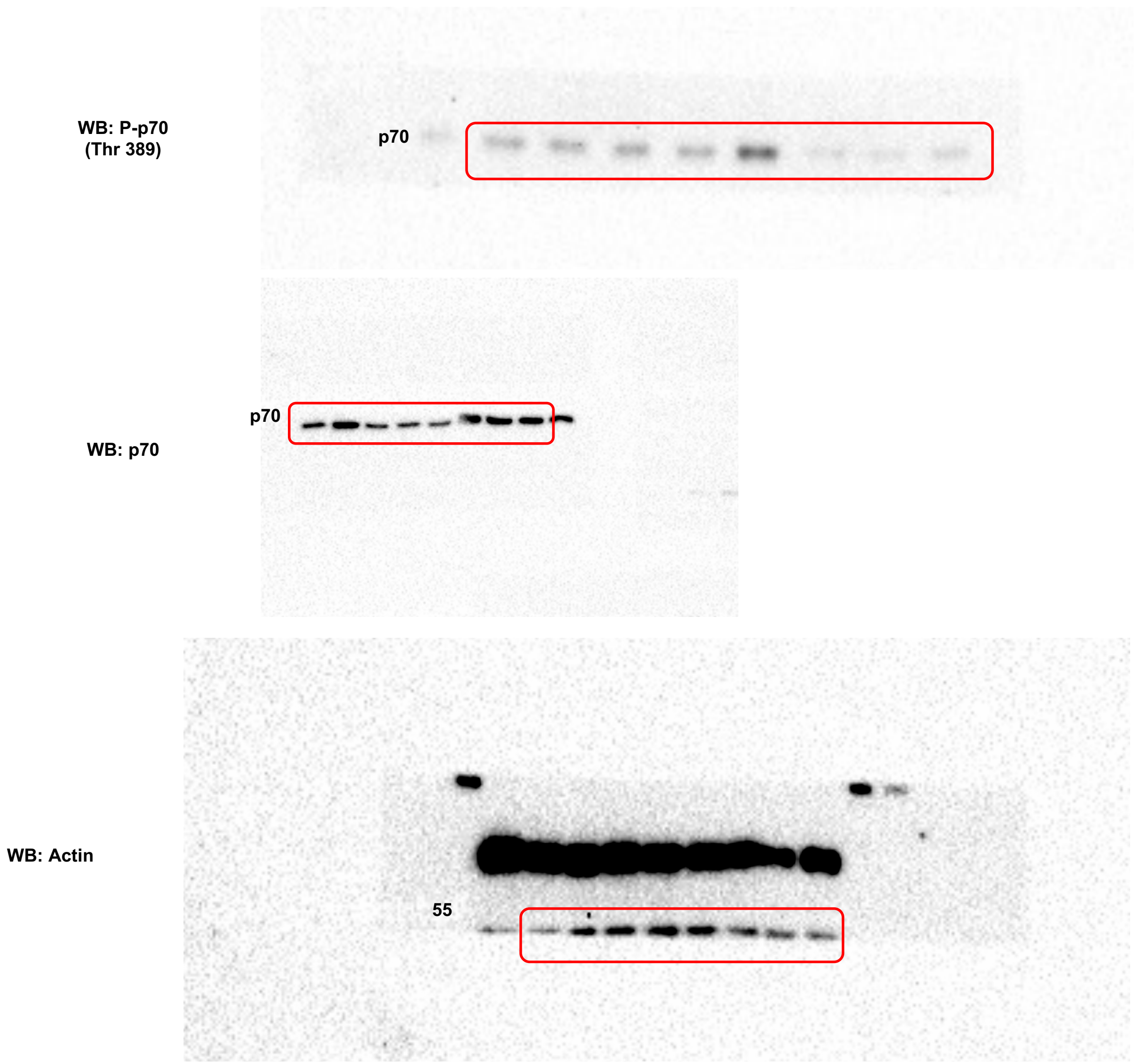

Suplemental Figure 3

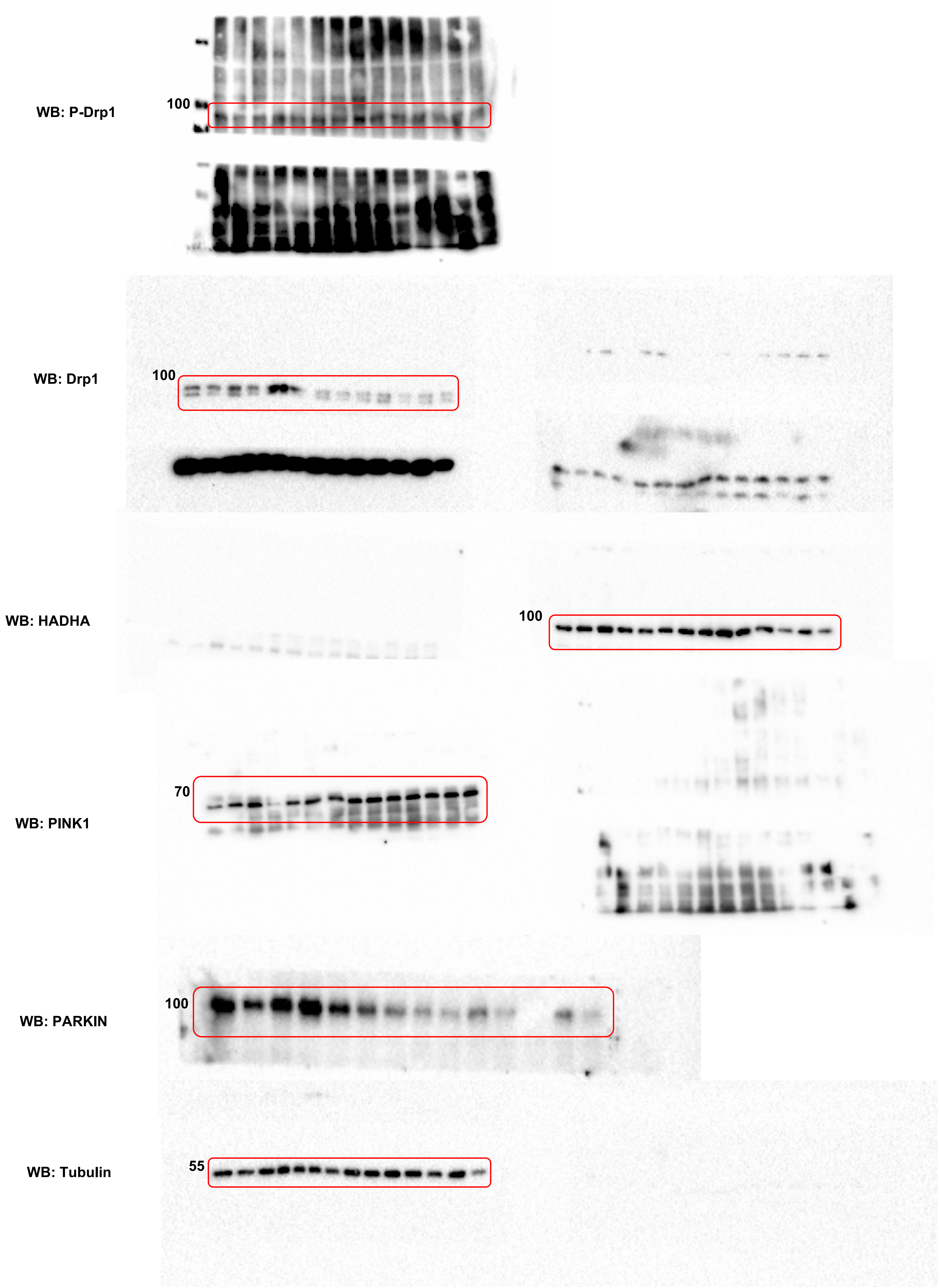

Supplement: Supplementary file 1 — Supplementary Information 1. [file 41598_2024_63525_MOESM1_ESM.pdf]
