# Supplementary material for: Regulation of TSC2 lysosome translocation and mitochondrial turnover by TSC2 acetylation status
Source: Sci Rep. 2024 May 31;14:12521. doi: 10.1038/s41598-024-63525-7 (PMC11143182; doi:10.1038/s41598-024-63525-7)
Supplement: Supplementary file 2 — Supplementary Information 2. [file 41598_2024_63525_MOESM2_ESM.pptx]

## Slide 1
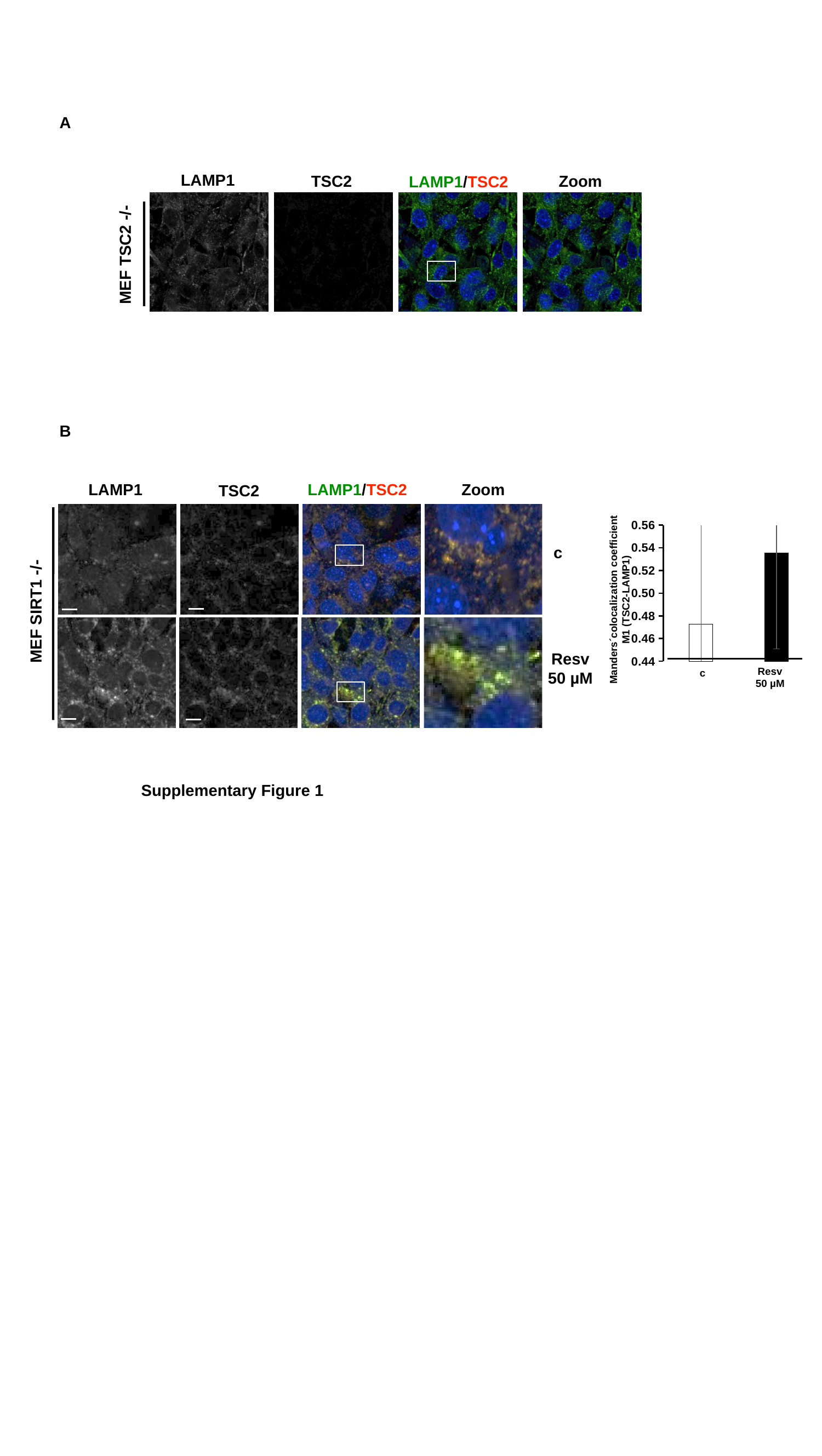

A
LAMP1
TSC2
Zoom
LAMP1/TSC2
MEF TSC2 -/-
B
LAMP1
LAMP1/TSC2
Zoom
TSC2
### Chart
| Category | |
|---|---|c
Manders´colocalization coefficient M1 (TSC2-LAMP1)
MEF SIRT1 -/-
Resv
50 µM
Resv
50 µM
c
Supplementary Figure 1

## Slide 2
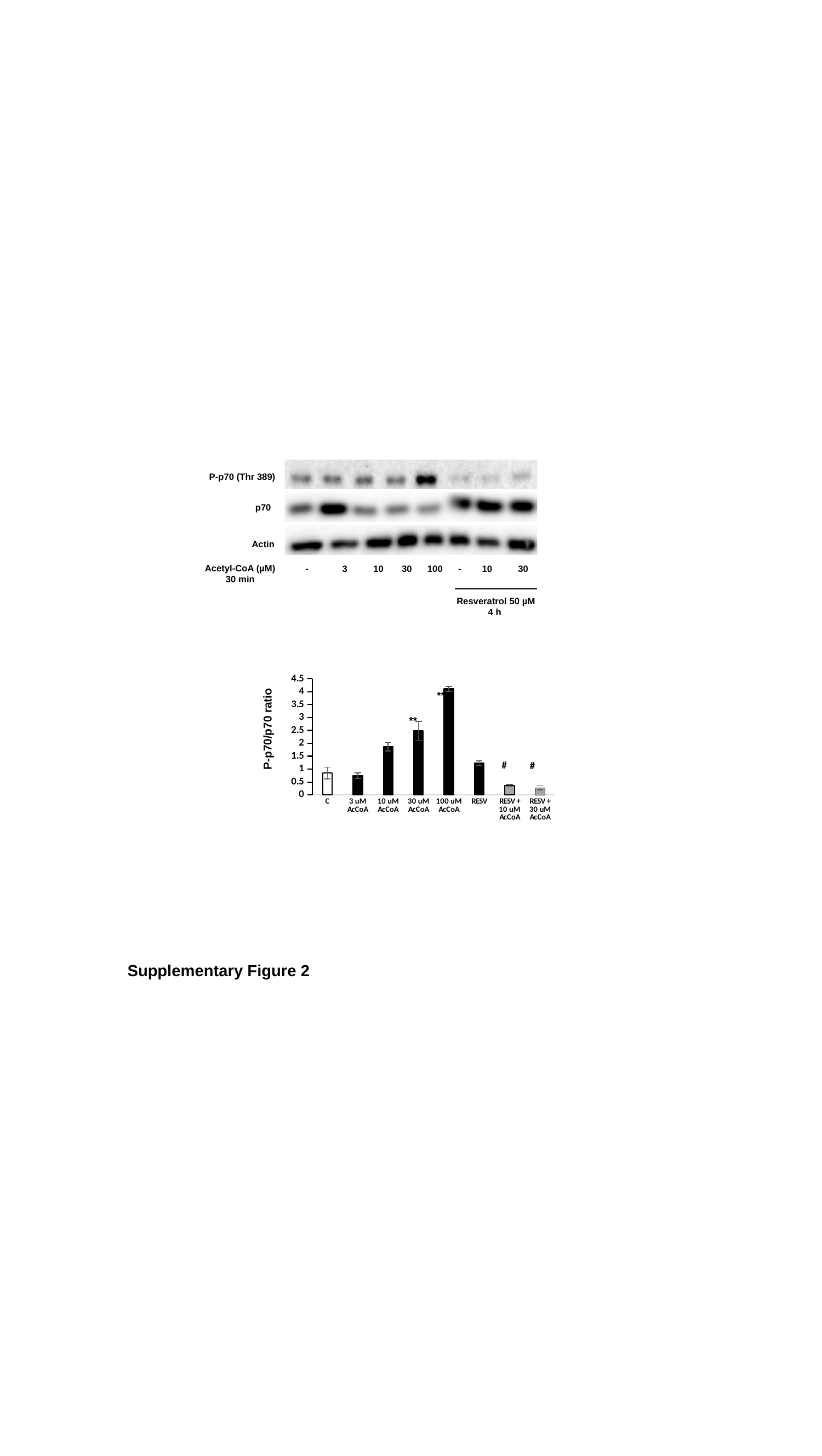

P-p70 (Thr 389)
p70
Actin
Acetyl-CoA (µM)
30 min
 - 3 10 30 100 - 10 30
Resveratrol 50 µM
4 h
### Chart
| Category | Ratio P-p70/p70 |
|---|---|
| C | 0.8476701874831664 |
| 3 uM AcCoA | 0.7465426325738732 |
| 10 uM AcCoA | 1.867999015731435 |
| 30 uM AcCoA | 2.4874601327250954 |
| 100 uM AcCoA | 4.11496062992126 |
| RESV | 1.2256409547670517 |
| RESV + 10 uM AcCoA | 0.36988937569170816 |
| RESV + 30 uM AcCoA | 0.27370176446620165 |***
**
P-p70/p70 ratio
#
#
Supplementary Figure 2

## Slide 3
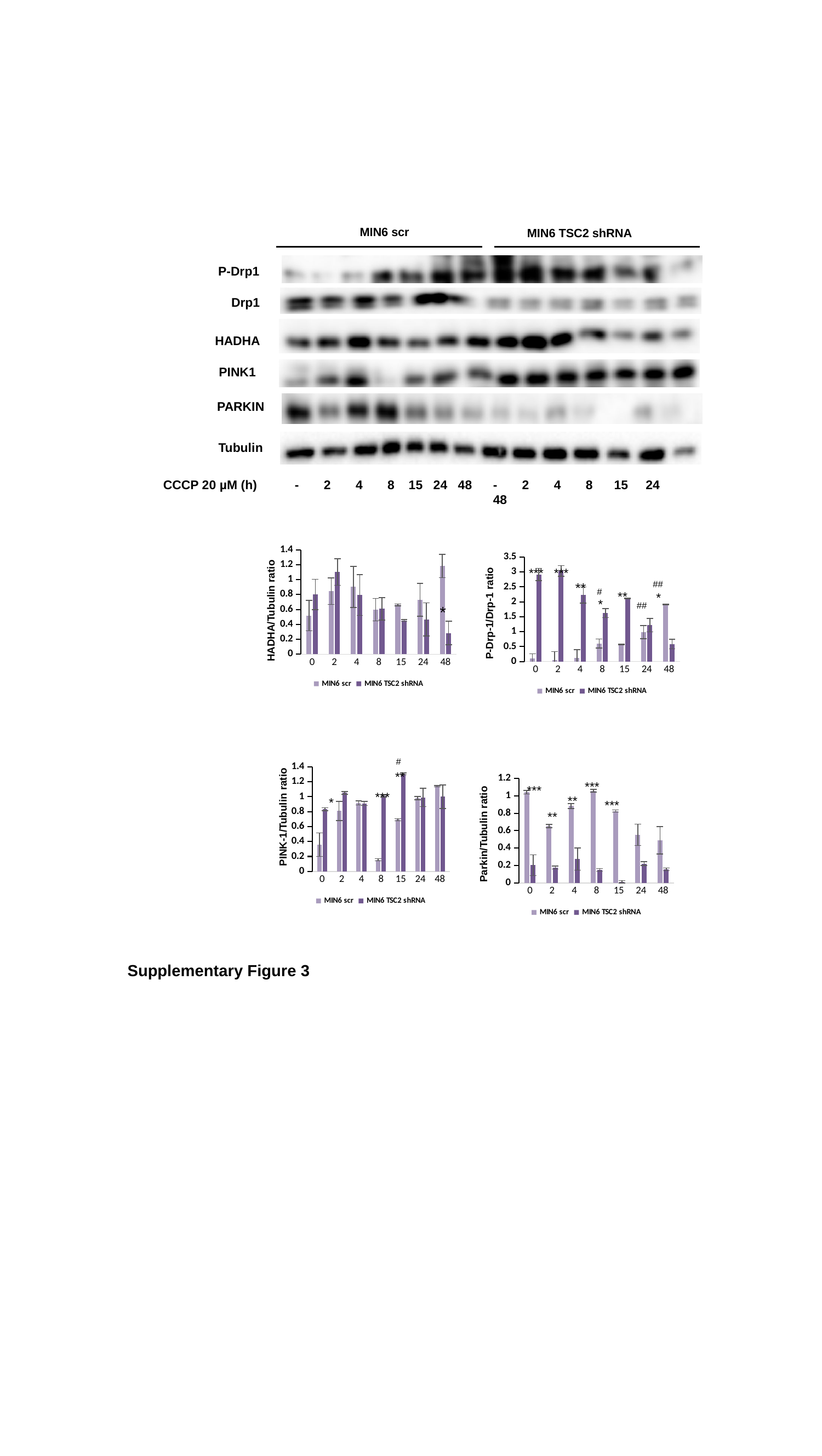

MIN6 scr
MIN6 TSC2 shRNA
P-Drp1
Drp1
HADHA
PINK1
PARKIN
Tubulin
CCCP 20 µM (h)
 - 2 4 8 15 24 48
- 2 4 8 15 24 48
### Chart
| Category | MIN6 scr | MIN6 TSC2 shRNA |
|---|---|---|
| 0 | 0.519273403697925 | 0.8021138368746367 |
| 2 | 0.8453356666230856 | 1.1024646565745027 |
| 4 | 0.9024914530651181 | 0.7937224230095377 |
| 8 | 0.5962069241770318 | 0.6098386038614685 |
| 15 | 0.6619027220421199 | 0.451732230696091 |
| 24 | 0.7300292148115324 | 0.4663377101279585 |
| 48 | 1.1829867313528684 | 0.28410136321819857 |*
### Chart
| Category | MIN6 scr | MIN6 TSC2 shRNA |
|---|---|---|
| 0 | 0.1029565924662708 | 2.9141830479813993 |
| 2 | 0.044760288196300756 | 3.034102847707138 |
| 4 | 0.11952958293802067 | 2.225369961143944 |
| 8 | 0.6012534002516566 | 1.6201426606944993 |
| 15 | 0.5650392593048792 | 2.108563957558347 |
| 24 | 0.9844076730804364 | 1.2168092926236516 |
| 48 | 1.9152839963519153 | 0.5861642654914895 |***
***
##
**
#
**
*
*
##
HADHA/Tubulin ratio
P-Drp-1/Drp-1 ratio
#
### Chart
| Category | MIN6 scr | MIN6 TSC2 shRNA |
|---|---|---|
| 0 | 0.3562467066350683 | 0.8332302667058722 |
| 2 | 0.809704172064437 | 1.0511924834455513 |
| 4 | 0.9185867707114933 | 0.9083224467482238 |
| 8 | 0.15557277226035762 | 1.0105643255312189 |
| 15 | 0.691853642105848 | 1.3068102482727897 |
| 24 | 0.9826451933913106 | 0.9911903298504405 |
| 48 | 1.1410045633154273 | 1.0 |**
***
*
### Chart
| Category | MIN6 scr | MIN6 TSC2 shRNA |
|---|---|---|
| 0 | 1.0424701289483023 | 0.203517339096377 |
| 2 | 0.6534668976568947 | 0.1767888674788028 |
| 4 | 0.8824989517282171 | 0.27408543299304 |
| 8 | 1.0573032441975223 | 0.15032502711220527 |
| 15 | 0.8255667780869473 | 0.013318113871087087 |
| 24 | 0.5519725493208947 | 0.22125493573564065 |
| 48 | 0.49074904475714903 | 0.15747020908866052 |***
***
**
***
**
PINK-1/Tubulin ratio
Parkin/Tubulin ratio
Supplementary Figure 3
