# Supplementary material for: Regulation of TSC2 lysosome translocation and mitochondrial turnover by TSC2 acetylation status
Source: Sci Rep. 2024 May 31;14:12521. doi: 10.1038/s41598-024-63525-7 (PMC11143182; doi:10.1038/s41598-024-63525-7)
Supplement: Supplementary file 3 — Supplementary Information 3. [file 41598_2024_63525_MOESM3_ESM.docx]

**Supplementary Figure 1**. A) Representative immunofluorescence images showing the negative signal of TSC2 in MEF TSC2 KO cells. B) Representative immunofluorescence images showing the reduction in the colocalization signal of TSC2 and LAMP1 in MEF SIRT1 -/- cells.

**Supplementary Figure 2**. Representative western blots showing the mTORC1 signaling pathways after the addition of a dose-response of acetyl-CoA in MIN6 Scr cells in the presence or in the absence of a pre-treatment with resveratrol at 50 µM. The graphs indicate the corresponding densitometric analysis and statistical analysis of either acetyl-lysine/Actin ratio or P-p70/p70 ratio. The values correspond to n=3. * One-way ANOVA was performed with Tukey's multiple comparisons test as *post hoc*; n=3. ** p ≤ 0,01 *** p ≤ 0,001 comparing MIN6 Tsc2 Scr treated with Ac-CoA vs control; ^#^ p≤0,05 ## p ≤ 0,01 MIN6 Tsc2 Scr treated with Ac-CoA + RESV vs RESV.

**Supplementary Figure 3**. The mitochondrial uncoupler CCCP stimulates mitophagy in MIN6 Scr pancreatic beta cells. Representative western blots of different mitophagy markers in MIN6 Scr and MIN6 Tsc2 shRNA pancreatic beta cells exposed to a time-course of CCCP 20 µM. The graphs correspond to the densitometric analysis of all the blots showing the mean ± SD, n=2. One-way ANOVA was performed with Tukey's multiple comparisons test as *post hoc*. * p ≤ 0,05 ** p ≤ 0,01 *** p ≤ 0,001 comparing MIN6 Tsc2 Scr vs Tsc2 shRNA; ^#^ p ≤ 0,05 ^##^ p ≤ 0,01 comparing either MIN6 Tsc2 Scr or Tsc2 shRNA treated with CCCP at different times with their respective controls at 0 hours.
